# Broken-fat pad sign: a characteristic radiographic finding to distinguish between knee rheumatoid arthritis and osteoarthritis

**DOI:** 10.1186/s13244-024-01608-9

**Published:** 2024-02-05

**Authors:** Qizheng Wang, Weili Zhao, Xiaoxi Ji, Yongye Chen, Ke Liu, Yupeng Zhu, Ruixin Yan, Siyuan Qin, Peijin Xin, Ning Lang

**Affiliations:** Department of Radiology, Peking University Third Hospital, 49 North Garden Road, Haidian District, Beijing, People’s Republic of China

**Keywords:** Magnetic resonance imaging, Infrapatellar fat pad, Rheumatoid arthritis, Osteoarthritis, Knee

## Abstract

**Objectives:**

Diagnostic imaging plays an important role in the pre-treatment workup of knee osteoarthritis (OA) and rheumatoid arthritis (RA). Herein, we identified a useful MRI sign of infrapatellar fat pad (IPFP) to improve diagnosis.

**Methods:**

Eighty-one age- and sex-matched RA and OA patients each, with pathological diagnosis and pre-treatment MRI were retrospectively evaluated. All randomized MR images were blinded and independently reviewed by two radiologists. The assessment process included initial diagnosis, sign evaluation, and final diagnosis, with a 3-week interval between each assessment. Broken-fat pad (BFP) sign was assessed on sagittal T2-weighted-imaging in routine MRI. The area under the curve and Cohen’s kappa (*κ*) were used to assess the classification performance. Two shape features were extracted from IPFP for quantitative interpretation.

**Results:**

The median age of the study population was 57.6 years (range: 31.0–78.0 years). The BFP sign was detected more frequently in patients with RA (72.8%) than those with OA (21.0%). Both radiologists achieved better performance by referring to the BFP sign, with accuracies increasing from 58.0 to 75.9% and 72.8 to 79.6%, respectively. The inter-reader correlation coefficient showed an increase from fair (*κ* = 0.30) to substantial (*κ* = 0.75) upon the consideration of the BFP sign. For quantitative analysis, the IPFP of RA had significantly lower sphericity (0.54 ± 0.04 vs. 0.59 ± 0.03, *p* < 0.01). Despite larger surface-volume-ratio of RA (0.38 ± 0.05 vs. 0.37 ± 0.04, *p* = 0.25) than that of OA, there was no statistical difference.

**Conclusions:**

The BFP sign is a potentially important diagnostic clue for differentiating RA from OA with routine MRI and reducing misdiagnosis.

**Critical relevance statement:**

With the simple and feasible broken-fat pad sign, clinicians can help more patients with early accurate diagnosis and proper treatment, which may be a valuable addition to the diagnostic workup of knee MRI assessment.

**Key points:**

• Detailed identification of infrapatellar fat pad alterations of patients may be currently ignored in routine evaluation.

• Broken-fat pad sign is helpful for differentiating rheumatoid arthritis and osteoarthritis.

• The quantitative shape features of the infrapatellar fat pad may provide a possible explanation of the signs.

• This sign has good inter-reader agreements and is feasible for clinical application.

**Graphical Abstract:**

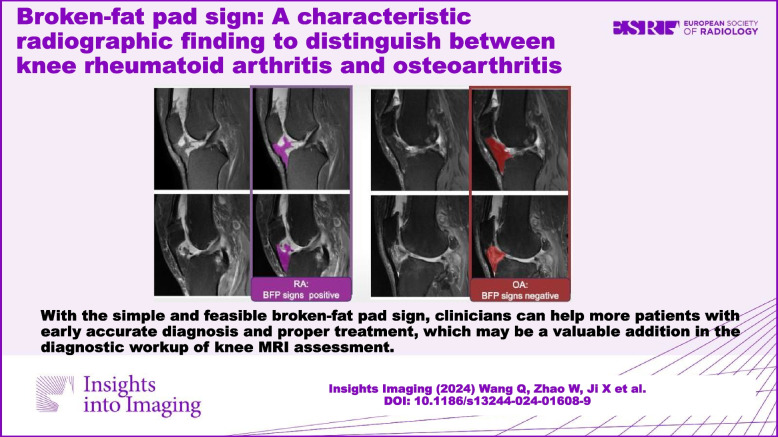

**Supplementary Information:**

The online version contains supplementary material available at 10.1186/s13244-024-01608-9.

## Introduction

Rheumatoid arthritis (RA) and osteoarthritis (OA) are characterized by joint destruction and inflammation [1]; however, there are highly overlapping features for both conditions through physical examination for both conditions and can be characterized by swelling, pain, effusion, and synovitis. Early classification of RA with knee joint involvement and knee OA is important because treatment and outcomes differ substantially [2].

MRI is increasingly being used for diagnosis in patients with knee symptoms; however, similar imaging findings such as cartilage defect, effusion, and synovitis have resulted in make an accurate differential diagnosis [3]. Many patients with a preliminary diagnosis of OA are discharged from the hospital for a routine follow-up with their primary care physician. This risks the possibility of a significant delay in the diagnosis of RA, which is detrimental to the patient’s joint function rescue. Despite recent advances in the pathophysiology of RA and OA [2, 4, 5], early diagnosis and therapeutic intervention remain challenging. Although biopsy-based analysis [6] and advanced MRI protocols [7] may be helpful for diagnosis, these modalities are often time-consuming, less accessible, and more expensive, which limits their routine use in clinics.

Concerning sources of intra-articular inflammation, infrapatellar (Hoffa’s) fat pad (IPFP) has become an area of intense research in recent years [1, 8, 9]. The presence of MRI alterations is common and can be well visualized in MR images, especially in the sagittal sequence. Recently, MRI evaluation of the subpatellar fat pad has attracted the attention of researchers. Most studies, whether by signal measurement [10], texture analysis [11], or functional MR imaging [12, 13], have focused only on OA; hence, only scarce information is available on IPFP in patients with RA. Furthermore, the technical threshold, such as advanced machine installation and higher level of technician and physician training, makes the clinical application difficult, especially in the common primary hospitals. To the best of our knowledge, there are no studies yet on the IPFP imaging changes for RA diagnosis.

Thus, the objective of our study was to determine the usefulness of morphological changes in IPFP to differentiate RA and OA of the knee joint based on conventional weighted MRI in a more generalizable manner.

## Materials and methods

### MR image datasets

The institutional review board of our institution approved this retrospective research (IRB00006761-M2023187), and the requirement for written informed consent was waived. A retrospective search of patients who underwent knee MRI for suspected RA or OA was conducted between 2012 and 2022.

The electronic medical records were reviewed for any history and direction of RA. Eighty-one patients who met the American College of Rheumatology/European League Against Rheumatism 2010 classification criteria [14] and/or the ACR 1987 criteria [15] for RA pathologically confirmed by arthroscopy were recruited.

These were then randomized with another 81 knee OA MRI images from age- and sex-matched patients to create a series for interpretation. Patients with knee OA were eligible for inclusion if they met the following three criteria: (i) preoperative MRI examination diagnosed knee OA, (ii) the ipsilateral knee underwent total joint replacement, and (iii) pathological diagnosis was chronic synovitis without clinical evidence of RA.

**Fig. 1:**
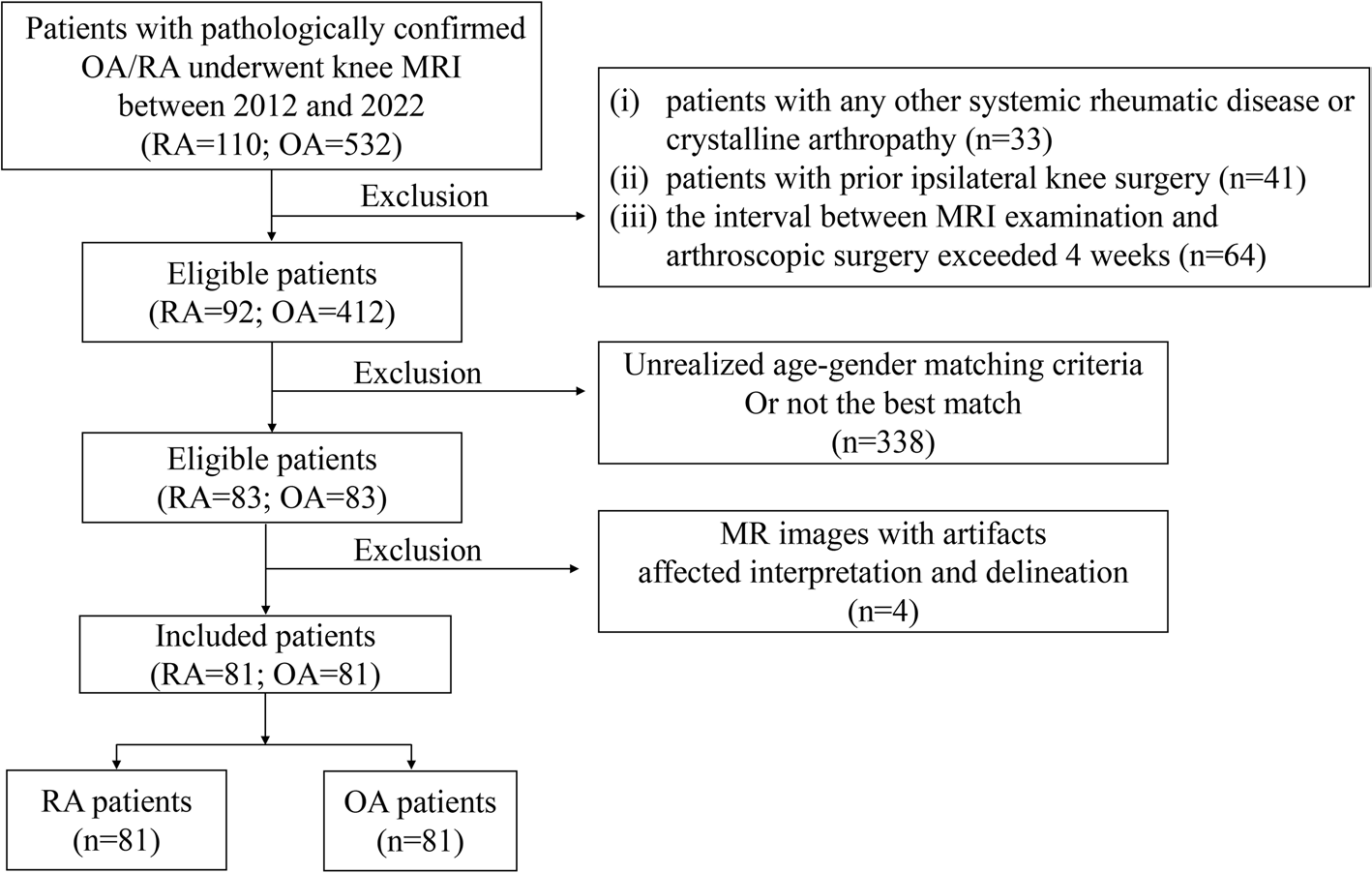
Flowchart of patient enrollment

All patients were excluded based on the following criteria: (i) presence of other systemic rheumatic disease or crystalline arthropathy, (ii) MR images with artifacts that affected interpretation and the region of interest delineation, (iii) patients with prior ipsilateral knee surgery, and (iv) the interval between MRI examination and arthroscopic surgery exceeded 3 weeks. Figure 1 shows a flow chart of patient enrollment.

### Image acquisition

Images were obtained on multiple MRI scanners, including three manufacturers and seven models (GE (Optima MR360, SIGNA Explorer, Signa, HDxt, Discovery, and MR750w), Siemens (Prisma), United Imaging United Imaging (uMR 780, uMR 660)), at either a 1.5-T or 3-T unit using a dedicated 8-/12-channel transmit/receive knee coil in neutral position. All studies included the following sequences: sagittal turbo or fast spin echo T1-weighted images and axial, coronal, and sagittal turbo or fast spin echo fat-saturated T2-weighted images. In our study, T2-weighted in sagittal plane images were obtained at a section thickness of 3–4 mm with a 1–2-mm intersection gap and a 16 × 16 field of view. Detailed parameters are presented in the Supplementary material.

### Image randomization and preprocessing

The routine knee MR images for all 162 patients were randomized and anonymized, completed by a physician (Z.W.). In the evaluation system, the patient’s name, ID, and disease diagnosis were blinded, but age and sex were retained. In addition, all images were cropped to show only the area around the subpatellar fat pad to minimize interpreter bias. The range was upper to the lower pole of the patella, lower edge to the tubercle of the tibia, anterior to the subcutaneous tissue, and posterior to the level of the tibial attachment site of the posterior cruciate ligament (the posterior margin of the tibial plateau). The cropped data will be used in sign evaluation, the second step of image interpretation.

### Image interpretation

**Fig. 2:**
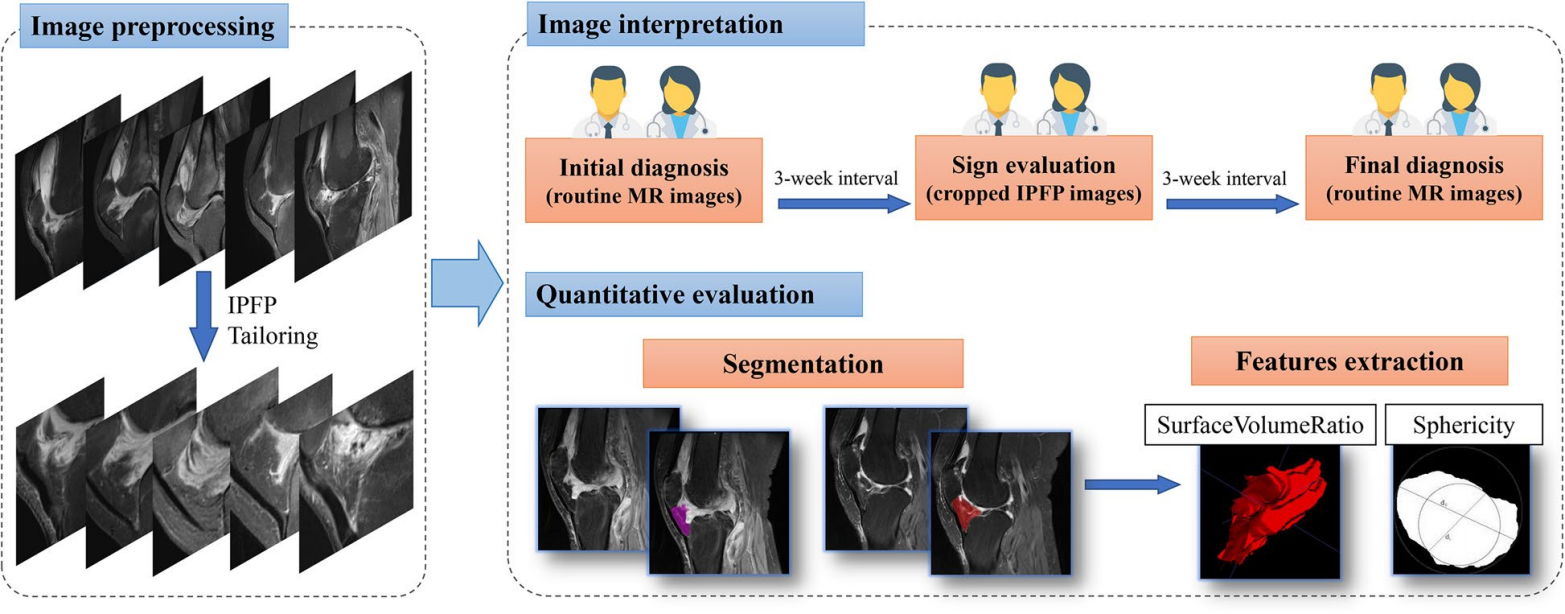
Flowchart of the study

The whole process of the evaluation consists of three stages (Fig. 2), including initial diagnosis (routine MR images), sign evaluation (cropped IPFP images), and final diagnosis (routine MR images). During the entire interpretation, two radiologists evaluated each patient independently and only the age and sex information were made known.

In the initial evaluation, all MR images (four routine sequences) of these 162 patients were independently reviewed by two radiologists (8 and 20 years in musculoskeletal imaging, respectively) blinded to clinical/pathological diagnosis. After the initial evaluation, the diagnosis was not disclosed to the two radiologists.

After 3 weeks, the cropped IPFP images were used to locate for the so-called broken-fat pad (BFP) sign. This sign was defined as the disappearance of the integrity of the IPFP and the emptiness of the fat pad area, over 1/2 of the posterior of the subpatellar fat pad by visual observation. On proton density weighted images of fat suppression images, the posterior margin of the IPFP with low signal was replaced by high signal, which usually showed a slit-like irregular shape with joint effusion. The significant discontinuity and irregularity of IPFP was defined as a positive BFP sign. Ten patients (BFP sign present: not present = 5:5) outside the paired cohort were used for training before formal interpretation. The definition of a training case was determined through a centralized discussion among five radiologists. The two radiologist readers in the image evaluation process are not included. We introduced only the semantic definition of the sign manifestation instead of angle or scale measurement to the two radiologists. Both of them only had to interpret the signs according to their understanding. The patients were classified according to the BFP sign in RA (positive sign) and OA (negative sign). Similarly, the correct diagnosis was not disclosed to either radiologist after the sign evaluation.

After a 3-week interval, the same two radiologists were asked to repeat the diagnosis based on the patient’s routine knee MRI image. The patient’s actual diagnosis and the initial diagnosis (6 weeks ago) were not visible. Prior to the assessment, the radiologists were informed that the BFP sign was more common in patients with RA. The two readers then made a final diagnosis based on the patient’s routine MR images.

### Segmentation and shape features extraction

For the sake of clinical interpretability, features descriptive of the IPFP shape were extracted to explore whether the MRI signs could be quantitatively explained. The region of interest was manually delineated according to the anatomical structure by a radiologist to generate a 3D mask of the infrapatellar fat pad. Features were extracted from each segmented volume using the PyRadiomics software package (version 2.2.0) after normalization. Instead of massive and complicated multidimensional features, two shape features were extracted for further analysis: surface-volume-ratio (the ratio of surface area to volume of a shape) and sphericity (a measure of how close the shape resembles a sphere). An example of delineation and feature interpretation diagrams is provided in Fig. 2.

### Statistical analysis

Patient demographics were summarized as mean with standard deviation for continuous variables and total with percentage for categorical variables. Using a binary diagnosis of RA versus OA, the diagnostic accuracy was summarized by calculating sensitivity and specificity, and 95% confidence intervals (CI) for sensitivity and specificity were calculated. Taking pathological diagnosis as the gold standard, the efficacies for the diagnosis of RA and OA were assessed by the receiver operating characteristic curve, and the area under the curve (AUC) was compared by DeLong’ test. Inter-reader agreement was assessed by using Fleiss’ *κ* with 95% CI. The kappa coefficient value was interpreted by Landis and Koch classification as follows [16]: ≤ 0.20, poor agreement; 0.21–0.40, fair agreement; 0.41–0.60, moderate agreement; 0.61–0.80, substantial agreement; and 0.81–1.00, almost perfect agreement. A two-sided *p* < 0.05 was considered to indicate statistically significant differences throughout the analysis. Statistical analysis was performed using SPSS (version 24.0, IBM Corporation, Armonk, NY, USA) and MedCalc (version 16.4.3, Ostend, Belgium).

## Results

### Patient demographics

A total of 162 patients were analyzed in this study. The study population comprised 34 male (21.0%) and 128 female (79%) subjects with a median age of 57.6 years (range: 31–78). There were 81 RA patients (56.62 ± 13.49 years) and 81 OA patients (56.01 ± 15.94 years). MRIs were performed on a 1.5-T magnet in 30 patients (18.5%) and on a 3.0-T magnet in 132 patients (81.5%). The interval between symptom onset and MRI examination ranged from 1 months to more than 10 years with a general history review. After knee joint symptoms, 43 (26.5%) people for inspection of patients within a year, 25 (15.4%) patients completed the examination in 1–3 years, and 73 (45.1%) patient completed knee MRI 3–5 years. Only 21 (13.0%) patients underwent knee examination more than 5 years after symptom onset.

### Sign evaluation and inter-reader agreement

**Fig. 3:**
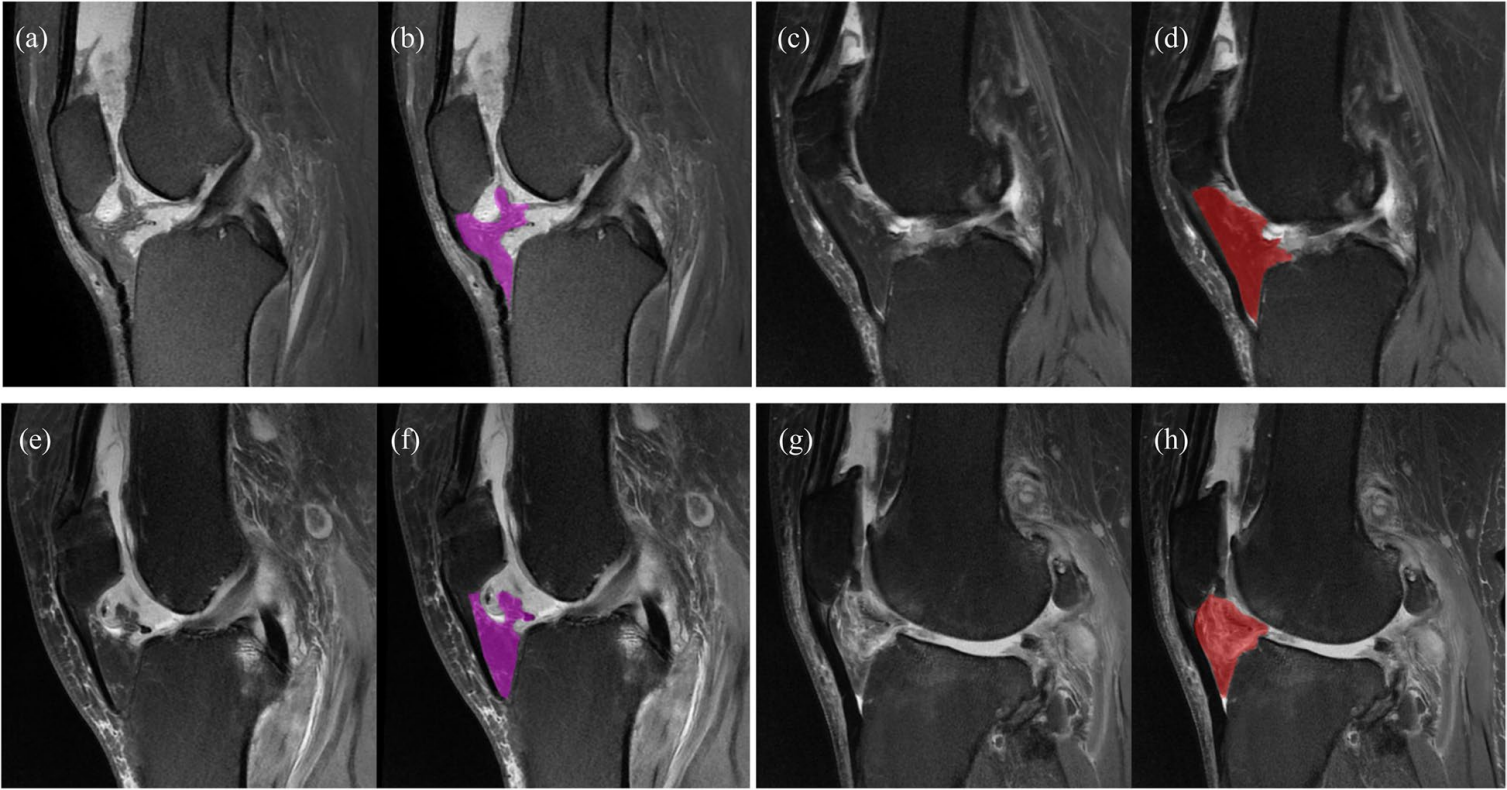
MRI images of two pairs of patients in our study cohort. The two aligned channels of four patient data (for visualization purpose, we only show the middle sagittal (mid-sagittal) slice of each channel. Purple and red masks are manual ROI for infrapatellar fat pad in patients with rheumatoid and knee osteoarthritis, respectively. **a**–**d** Two 54-year-old male patients with knee pain. **a**, **b**: RA showed positive broken-fat pad (BFP) sign. **c**, **d** OA showed negative BFP. **e**–**h** Two 66-year-old female patients with limited knee function. RA patients (**e**–**f**) showed positive BFP signs, and OA patients (**g**–**h**) showed negative signs

Of the study population, 59 (59/81, 72.8%) patients had BFP sign in our cohort of RA patients according to the interpretation of the senior radiologist (reader 2). The proportion of positive signs was much higher (*p* < 0.001) than that in the osteoarthritis cohort (17/81, 21.0%). Figure 3 shows image examples of patients with/without the BFP sign. With regard to the sign evaluation, there was substantial agreement (kappa coefficient: 0.73, 95% CI: 0.62–0.83) between the junior and senior radiologists. Combined with the positive signs for the diagnosis, the agreement between radiologists improved conspicuously, from fair (*κ* = 0.30, 95% CI: 0.15–0.44) to substantial (*κ* = 0.75, 95% CI: 0.65–0.85). Detailed results are presented in Table 1.

**Table 1:** Inter-reader agreement for RA cases detected in different stages

Evaluation process	RA cases detected by observers		Inter-reader agreement
	Reader 1	Reader 2	
Initial diagnosis	71 (43.8%)	69 (42.6%)	0.30 (0.15–0.44)
Sign evaluation	72 (44.4%)	76 (46.9%)	0.73 (0.62–0.83)
Final diagnosis	78 (48.1%)	80 (49.4%)	0.75 (0.65–0.85)

### Diagnostic performance

**Fig. 4:**
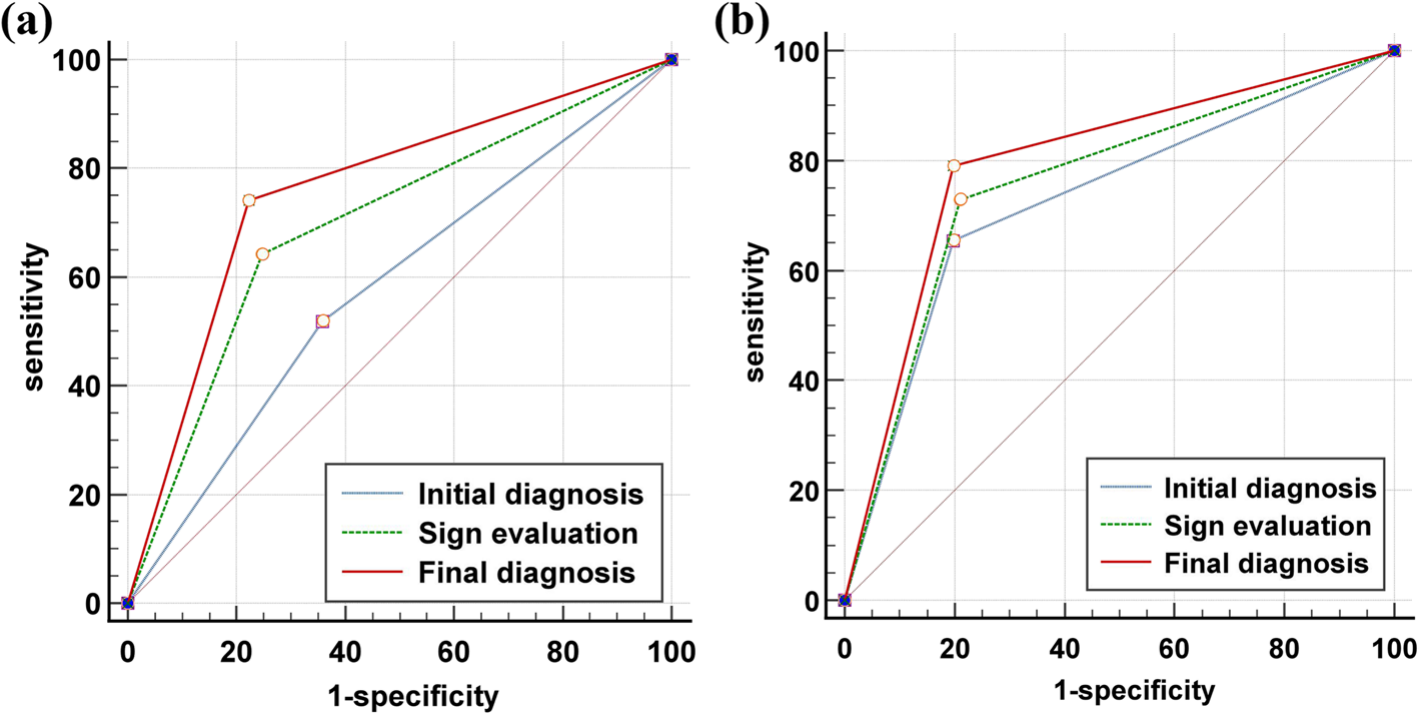
Receiver operating characteristic analysis of the two radiologists and each evaluation process. **a** Reader 1 with 8 years’ experience, with AUC increased from 0.58 to 0.76. **b** Reader 2 with 20 years’ experience, with AUC increased from 0.73 to 0.80. AUC, area under the curve

In the three stages of evaluation, the diagnostic accuracy of the junior (reader 1) and senior (reader 2) readers was improved (reader 1: 0.58 vs. 0.70 vs. 0.76; reader 2: 0.73 vs. 0.76 vs. 0.80), especially the sensitivity of detecting knee RA was significantly improved (reader 1: 0.52 vs. 0.64 vs. 0.74; reader 2: 0.65 vs. 0.73 vs. 0.79). A summary of the diagnostic performance is provided in Table 2. For reader 1, the AUC of the initial diagnosis and final diagnosis was statistically different (0.58 vs. 0.76, *p* = 0.0001, Delong test). For reader 2, the AUC of sign evaluation and final diagnosis was statistically different (0.76 vs. 0.80, *p* = 0.0314, Delong test). Figure 4 shows the diagnostic performance of radiologists and the evaluation process.

**Table 2:** Diagnostic performance of radiologists and evaluation process

	TP	FP	FN	TN	Accuracy (%)	Sensitivity (%)	Specificity (%)	AUC	SE	95% CI
**Reader1**										
Initial diagnosis	42	29	39	52	58.02	51.85	64.20	0.58	0.039	0.50–0.66
Sign evaluation	52	20	29	61	69.75	64.20	75.31	0.70	0.036	0.62–0.77
Final diagnosis	60	18	21	63	75.93	74.07	77.78	0.76	0.034	0.69–0.82
**Reader2**										
Initial diagnosis	53	16	28	65	72.84	65.43	80.25	0.73	0.035	0.65–0.80
Sign evaluation	59	17	22	64	75.93	72.84	79.01	0.76	0.034	0.69–0.82
Final diagnosis	64	16	17	65	79.63	79.01	80.25	0.80	0.032	0.73–0.86

*TP*, True-positive, *FN* False-negative, *TN* True-negative, *FP* False-positive, *AUC* Area under the curve, *SE* Standard error, *CI* Confidence interval

### Quantitative analysis based on IPFP morphology

**Fig. 5:**
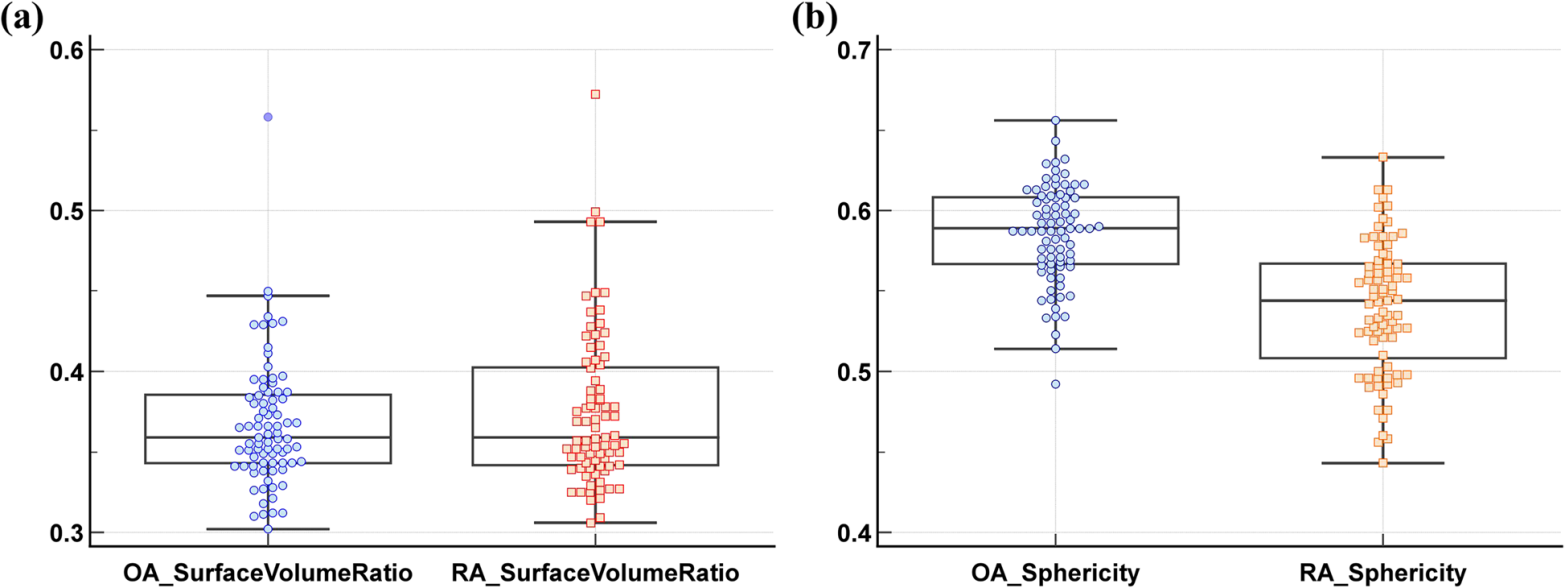
Quantitative features of IPFP morphology. **a** Surface-to-volume ratio. **b** Sphericity. The blue and orange markers are for osteoarthritis and rheumatoid arthritis, respectively

For quantitative analysis based on IPFP, univariate analysis showed that IPFP of RA patients had significantly lower sphericity (0.54 ± 0.04 vs. 0.59 ± 0.03, *p* < 0.0001) and larger surface-volume-ratio with no statistical difference (0.38 ± 0.05 vs. 0.37 ± 0.04, *p* = 0.2515) than OA patients. The center-specific medians, means, lower, and upper quartiles and outliers of each feature are shown in Fig. 5.

## Discussion

The broken-fat pad sign is reliable, sensitive, and specific for the detection of RA based on routine MRI. In this study, based on proton density weighted imaging (PDWI) images in the routine clinical examination pathway, the diagnostic efficacy and inter-reader agreement of this sign were explored. This cost-saving and practical method would be a useful adjunct in cases where the clinical findings are equivocal. A prompt differential diagnosis with simple methods plays a crucial part in optimizing return of knee joint function.

Although MRI is increasingly used in patients with knee symptoms and provides a remarkable value for differential diagnosis, it was found that OA is one of the most common causes of equivocal diagnosis of RA [17]. This implies significant differential diagnostic challenges in the clinical pathway based on the same radiographic findings (synovial hyperplasia, osteochondral injury, effusion, and edema) in clinical practice, especially in primary care centers. It is now well-understood that delay of disease-modifying anti-rheumatic drug (DMARD) therapy in RA is a major contributing factor for poor outcome [18].

Regarding the origin of intra-articular inflammation, IPFP, also known as Hoffa’s fat pad, is considered an emerging player [19]. There are studies that support the emerging idea that the IPFP and synovium may be considered as an anatomic-functional unit [20]. Therefore, some advanced MRI imaging methods have been used to investigate IPFP lesions, such as contrast-enhanced MRI [12], modified DIXON sequences [21], and double-echo in steady-state sequence [22]. However, advanced enhanced scanning or advanced sequence has high cost and application threshold, especially in most primary hospitals, and is not widely used at present. To our knowledge, no study yet has paid attention to the differences in imaging manifestations of IPFP between RA and OA patients in routine PDWI examination. Therefore, our study focused on the MRI signs of IPFP in the routine medical plan for the differential diagnosis of RA and OA. Our proposed method considered the following criteria: (a) it should be accurate and simple; (b) it should not be strictly related to the scanner, field strength, and knee flexion; (c) its repeatability should be independent of patient characteristics; and (d) inter-reader agreement should be acceptable.

Our study found that morphological changes of IPFP observed on MRI had high inter-reader agreement and optimal diagnostic performance. Broken-fat pad sign is more common in patients with RA (68.52%) than OA (22.84%), which may be because of a more intense inflammatory mechanism in RA. By recognizing this simple sign, physicians, especially non-expert readers, can significantly improve the diagnostic efficacy. Equally important, the duration of examination in our cohort ranged from 6 months to more than 10 years after symptom onset, suggesting that this sign may be reported at various stages of the disease course. If the physician considers the differential diagnosis of RA and OA in patients with knee symptoms, an earlier MRI may be recommended. It is reasonable to consider that IPFP shape is important, especially because it has not previously been defined in an appropriate manner to determine the true diagnosis value.

Observable inter-patient heterogeneity exists in radiological IPFP appearance [23, 24]. Therefore, we have not attempt to quantify the actual size of the IPFP, but rather its shape. Through quantitative analysis based on feature extraction, the surface area to volume ratio and sphericity may provide a quantitative approach for the morphological characterization of IPFP. Our results suggest that the IPFP of RA had significantly lower sphericity (0.54 ± 0.04 vs. 0.59 ± 0.03, *p* < 0.01) than OA conducted by 3D mask, which to some extent contributes to the interpretation of BFP signs. Quantitative extraction of shape features provided a more objective definition of IPFP, but the technical threshold is high (multi-layer manual delineation, embedded analysis software, etc.). Thus, we believe that the BFP sign may be helpful and practical for radiologists to detect RA without additional facilities or assessment burden in daily work, especially in junior radiologists. Although it is subjective, it is a diagnostic sign that can be easily generalized with acceptable inter-reader agreement in our study.

While the BFP sign demonstrated promising results, we acknowledge that our study had some limitations. First, we did not conduct a matched pair study based on weight. Though previous studies have suggested that body weight may have an effect on IPFP, this remains controversial [23, 24]. Second, it is difficult to completely exclude all bones in IPFP image cropping, which may cause potential interference. Third, a larger prospective study of this criterion is needed to further demonstrate the reliability of the sign. We expect that this criterion combined with detailed patient history, skilled physical examination, and multitype imaging findings, will substantially diminish the incidence of missed patients with combined knee RA and reduce the need for more expensive tests such as enhanced MRI. It is important to note that the absence of this sign should not supersede other suggestive signs of RA.

Our results suggest that the BFP sign is a strong radiological indicator suggestive of knee RA, which may contribute to early treatment. The attention to morphological changes in the IPFP may increase the usefulness of routine MRI in the differential diagnosis of OA and RA.

### Supplementary Information

**Additional file 1: Table S1. **MRI sequence parameters used in the study.

## Data Availability

The data supporting the findings of this study are available within the article and its supplementary materials.

## Abbreviations

AUC: Area under the curve
BFP: Broken-fat pad
CI: Confidence intervals
IPFP: Infrapatellar fat pad
OA: Osteoarthritis
RA: Rheumatoid arthritis
