# Supplementary material for: Broken-fat pad sign: a characteristic radiographic finding to distinguish between knee rheumatoid arthritis and osteoarthritis
Source: Insights Imaging. 2024 Feb 5;15:33. doi: 10.1186/s13244-024-01608-9 (PMC10844185; doi:10.1186/s13244-024-01608-9)
Supplement: Supplementary file 1 — Additional file 1: Table S1. MRI sequence parameters used in the study. [file 13244_2024_1608_MOESM1_ESM.docx]

**Broken-fat pad sign: A characteristic radiographic finding to distinguish between knee rheumatoid arthritis and osteoarthritis**

**ELECTRONIC SUPPLEMENTARY MATERIAL**

Table S1. MRI sequence parameters used in the study.

|  | Manufacturer | | | | | | |
| --- | --- | --- | --- | --- | --- | --- | --- |
|  | GE | GE | GE | GE | Siemens | United Imaging | United Imaging |
| Model Name | Optima MR360 | SIGNA Explorer | Signa HDxt | Discovery MR750w | Prisma | uMR 780 | uMR 660 |
| Magnetic Field Strength | 1.5T | 1.5T | 3.0T | 3.0T | 3.0T | 3.0T | 1.5T |
| Repetition time, ms | 2494 (391) | 2940 (451) | 2460 (420) | 2909 (732) | 2900 (576) | 2682 (525) | 2746 (552) |
| Echo time, ms | 35 (14) | 39 (10) | 38 (13) | 34 (8) | 35 (11) | 36 (9) | 41.2 (10) |
| Echo train length | 10 (3) | 10 (3) | 10 (3) | 8 (3) | 7 (3) | 6 (3) | 7 (3) |
| Pixel Bandwidth, Hz | 122 (109) | 81 (98) | 122 (122) | 122 (163) | 160 (305) | 180 (250) | 150 (200) |
| Flip angle, degrees | 90 | 90 | 90 | 90 | 90 | 90 | 90 |
| Field of view, mm | 160×160 | 160×160 | 160×160 | 160×160 | 160×160 | 160×160 | 160×160 |
| Slice thickness, mm | 3.5 | 3.5 | 3.5 | 3.5 | 3.5 | 3.5 | 4.0 |

Note: proton density weighted sequence parameters ant T1 weighted sequence parameter, the latter in parentheses.
